# Interaction of Sensitizing Dyes with Nanostructured TiO_2_ Film in Dye-Sensitized Solar Cells Using Terahertz Spectroscopy

**DOI:** 10.1038/srep30140

**Published:** 2016-07-22

**Authors:** William Ghann, Aunik Rahman, Anis Rahman, Jamal Uddin

**Affiliations:** 1 Center for Nanotechnology for Department of Natural Sciences, Coppin State University, 2500 W. North Avenue, Baltimore, MD 21216, USA; 2 Applied Research & Photonics, 470 Friendship Road, Suite 10, Harrisburg, PA 17111, USA.

## Abstract

The objective of this investigation was to shed light on the nature of interaction of different organic dyes and an inorganic dye, Ruthenium (II) polypyridine complex, with TiO_2_ nanoparticles. TiO_2_ is commonly deployed as an efficient energy transfer electrode in dye sensitized solar cells. The efficiency of dye sensitized solar cells is a function of the interaction of a dye with the electrode material such as TiO_2._ To the best of our knowledge the present study is the first effort in the determination of terahertz absorbance signals, investigation of real-time dye permeation kinetics, and the surface profiling and 3D imaging of dye sensitized TiO_2_ films. Herein, we report that the terahertz spectra of the natural dye sensitized TiO_2_ films were distinctively different from that of the inorganic dye with prominent absorption of natural dyes occurring at approximately the same wavelength. It was observed that the permeation of the natural dyes were more uniform through the layers of the mesoporous TiO_2_ compared to the inorganic dye. Finally, defects and flaws on TiO_2_ film were easily recognized via surface profiling and 3D imaging of the films. The findings thus offer a new approach in characterization of dye sensitized solar cells.

Terahertz radiation (T-ray) based spectroscopy employs a range of wavelengths between the microwave and the far-infrared region, from ~10 μm to ~3000 μm. Transient kinetics and different molecular resonances such as vibrational states of materials may be probed with T-rays^1^,^2^,^3^. Terahertz time-domain spectroscopy (THz-TDS) has recently gained attention as a valuable tool for probing molecular transitions that are usually not captured by other spectroscopic techniques such as UV, infrared and Raman spectroscopy^4^,^5^,^6^. The non-ionizing and noninvasive nature of the radiation coupled with its relative transparency to most materials except metals makes it suitable for the analysis of biological structures in their native state.

Another major benefit of THz-TDS is time resolution, which is very important to the dynamics of certain processes, allowing photo-induced responses to be characterized with sub-picosecond temporal resolution^7^. Terahertz spectrometry has been used in a number of applications including the investigation of the permeation kinetics and concentration profile of active ingredients into the human stratum corneum^8^, as well as other biomedical^9^,^10^, pharmaceutical^11^, proteomics^12^,^13^ and genomics studies^12^,^13^. Terahertz spectrometry has also been used in the sensing and identification of explosives and other materials of security concerns^14^.

The terahertz portion of the electromagnetic radiation is very sensitive to detailed structural properties as well as charge migration and in recent times have been used to study the photoconductivity and the dynamics of charge separation of several photovoltaic devices^7^,^10^,^15^,^16^,^17^. Dye sensitized solar cells (DSSC) are a class of photovoltaic devices that have been widely investigated due to advantages such as ease of fabrication, low cost, ecofriendly nature and appreciable solar-to-electric energy conversion efficiency^18^,^19^,^20^. DSSC comprises of a photoanode, an electrolyte system for charge regeneration and a counter electrode. The present work focuses on the photoanode which consist of a transparent conducting oxide (typically Indium doped Tin Oxide, ITO, or Fluorine doped Tin Oxide, FTO) substrate covered with a thin film of titanium dioxide (TiO_2_) nanoparticles, and a dye^21^. The dye is used to sensitize the wide band gap TiO_2_ electrode. Upon illumination by solar energy, dye molecules absorb photons and move to the excited states. The excited electrons are subsequently injected into the TiO_2_ conduction band. An injected electron diffuses through the nanocrystalline TiO_2_ to the conductive film and then transferred to an external circuit^22^,^23^,^24^. An in-depth understanding of the interaction of dyes with the electron-transporting TiO_2_ electrodes will form the basis for further research that will improve our understanding of the mechanisms of charge generation and transport leading up to the production of solar energy. A number of studies have been carried out to investigate the influence of dye sensitization on the overall efficiency of solar cells^25^,^26^,^27^,^28^.

In the present paper, T-rays have been used to investigate the interaction properties of the photoanode component of the solar cell and could provide information on any defects that can potentially affect the overall efficiency of dye sensitized solar cells. The TiO_2_ film provides an efficient scaffold to hold large amount of dye molecules needed for light harvesting. However, if there are cracks in this scaffold, it reduces the number of dye molecules adsorbed on the TiO_2_ and thus subsequently affect the efficiency of the solar cell. A means to detect cracks and flaws in the TiO_2_ film and consequently the dye sensitized solar cell will thus help improve the fabrication of high performance dye sensitized solar cells. The three dyes chosen for the studies were two different organic (pomegranate and blackberry dye) and an inorganic dye, Ruthenium (II) polypyridine complex (Rubpy). The three dyes were chosen mostly on the account of their strong absorption properties. Rubpy complexes are the most common type of dye utilized in the fabrication of dye sensitized solar cells^29^. Blackberry and Pomegranate, like other natural dyes used in solar cell fabrication, have appreciable light absorption properties and preferable in terms of their ecofriendly nature, low cost and abundance^30^,^31^,^32^. The various anchoring groups on the dyes provide means of attachment to the TiO_2_ surface. Herein is reported terahertz reflectometry and spectrometry studies on dye sensitized titanium dioxide coated FTO glass plates commonly used in the fabrication of DSSC. Terahertz measurements were carried out using a terahertz time-domain spectrometer with an attached nanoscanner as displayed in Fig. 1. The spectral features of the dyes adsorbed on TiO_2_ were studied using THz-TDS techniques and its characteristic spectra ranging from 0 to 5 THz were obtained. A fast and effective methodology was developed for the inspection and identification of defects on solar cells.

## Results and Discussion

### Spectroscopic Studies of Dye Sensitized TiO_2_ films

Teraspectra has been shown to be very sensitive to various resonances in number of different molecules^4^,^5^. Many materials exhibit distinct spectral absorption features in the THz range of the electromagnetic spectrum and this makes possible the identification and characterization of such materials^33^,^34^. Terahertz radiation is sensitive to the vibrational states of an entire molecule and terahertz spectra, accordingly, corresponds to molecular or inter-molecular behavior and provides information unique to a given molecule or substance. As a result, no two molecules exhibit exactly identical terahertz absorbance peaks as it is in the case of mid-infrared spectra which give intra-molecular information^5^. Light absorption by dye sensitized TiO_2_ coated FTO plates were investigated with the terahertz probe pulses. The terahertz characteristic absorption spectra were obtained in the range of 0–35 THz. However, for the reason that too many peaks placed on a single plot makes the plot very busy, only data in the range of 0–5 THz are reported in this paper. The THz-TDS spectrometer generates data first in the time domain to which Fourier transform algorithms is applied to obtain a frequency spectrum. Figure 2a–c exhibits the time-domain pulses (or interferogram) of the three dyes studied. Although T-ray provides information about the entire molecule it could be seen that the interferogram of the natural dyes (Fig. 2a,b) follow the same trend as opposed to the inorganic dye. The time-domain signal in Fig. 2a–c were Fourier transformed to obtain the terahertz spectra in Fig. 2d–f. The terahertz spectral features obtained for the natural dyes were very similar to each other and had a lot of overlapping peaks when the three spectra where displayed together in Fig. 3.

Pomegranate dye sensitized TiO_2_ films exhibited prominent absorption peaks at 0.33 THz, 0.97 THz, 1.99 THz, 2.85 THz, 3.54 THz and 4.48 THz. Blackberry dye sensitized TiO_2_ on the other hand exhibited prominent absorption peaks at 0.54 THz, 1.3 THz, 2.0 THz, 2.8 THz, 3.5 THz and 4.5 THz. The overlay of the spectra of all three dyes in Fig. 3 shows that the absorption characteristics of the natural dyes are very similar: the absorption peaks overlap with each other and are much sharper than the peaks of the Rubpy sensitized films. Rubpy sensitized TiO_2_ films had only high absorption peak at 0.97 THz which was comparable to that of pomegranate and blackberry dye sensitized TiO_2_ films.

### Kinetic Studies of Dye Sensitized TiO_2_ films

Kinetic studies were conducted on the three dye sensitized TiO_2_ films to evaluate the rate at which the dyes diffuse through the TiO_2_ nanoparticle matrix. Aliquot 20 μL of the dye was dropped on the film and the transient kinetics of diffusion of the dyes was recorded over a period of 500 seconds as the dyes permeated the titanium dioxide film. Figure 4 exhibits the characteristics of diffusion of the respective dyes in titanium dioxide substrate. With all the dyes, there is an initial broad decrease in intensity counts followed by an increase in intensity counts before they level off. Based on the slope of the curves in the first 100 seconds, Rubpy seems to have the fastest kinetics, reaching the peak of its intensity counts within 40 seconds after the initial drop in intensity. After this increase in transmitted intensity, there is a gradual decrease in intensity until it reaches a plateau. Rapid evaporation of the dye during the measurement, which most likely exposes some part of the titanium dioxide nanoparticles, could account for this sudden increase in intensity. Unlike pomegranate and blackberry dyes which were used in their natural form, Rubpy solution was prepared by dissolving the solid form in a suitable amount of deionized water and this could account for the rapid diffusion rate. After the initial decrease in intensity upon application of the pomegranate dye, there is a gradual increase in intensity from 12 seconds after dye application to about 100 seconds after dye application. The intensity counts subsequently decreases till it levels off. Blackberry dye has the slowest kinetics; after the initial drop in intensity counts upon application of dye, it takes over 100 seconds to reach the maximum intensity and then steadily decrease in intensity till it reaches a plateau. Rubpy diffusion reached saturation faster than both of the natural dyes as indicated by flattening of its slope. Figure 4d–f shows a close-up of Fig. 4a–c where the X-axis is zoomed to 100 seconds. It can be observed that the inorganic dye (Rubpy) shows an initial transmission count higher than that of FTO/TiO_2_ substrate alone whereas pomegranate and blackberry show a decrease in intensity before slowly going up in intensity count. This may be indicative of the fact that each of these dyes interact with the TiO_2_ nanoparticles in their own unique way. Further investigation will be conducted for conclusive remarks on this observation.

### Depth Scan Measurement of Dye Sensitized TiO_2_ films

The Terahertz scanning reflectometry was used to assess the concentration profile of the different dyes into the TiO_2_ film. The technique has been previously used to investigate the concentration profile of the active ingredients across the stratum corneum of the skin^8^. Depth scan of TiO_2_ films before and after saturation with the various dyes was carried out to assess their reflectance at increasing depth and thereby understand the distribution of the dye in the titanium dioxide mesoporous film. The scanning measurement was first carried out on the blank FTO/TiO_2_ film, as a control, after which 20 μL of dye was applied and allowed to fully saturate the TiO_2_ layer. All the measurements were carried out under identical conditions. A second scanning measurement was carried out after TiO_2_ was fully saturated with the dye. The film was considered fully saturated when kinetics of the dye reached a steady state. The difference in reflectance intensity of the FTO/TiO_2_ film saturated with the dye offers insight into the concentration of the dyes across the depth of the TiO_2_ film. The intensity of terahertz radiation through a portion of the glass substrate was also measured since it is dependent on the amount of light adsorbed in the Dye/TiO_2_ film. Figure 5 (inset) shows the computation of the amount of Rubpy dye (green curve) from the scan of the blank (red curve) and saturated substrate (blue curve). The result of the measurements as displayed in Fig. 5 show that the concentration of Rubpy in the TiO_2_ is not uniform indicated by the increase in intensity with increasing depth of the mesoporous film. Conversely, the permeation of the natural dyes are uniform through all the layers of the mesoporous TiO_2_ film. The concentration of blackberry dye is, however, relatively more constant through the layers. This result corroborates with that of the kinetic study (Fig. 4) which shows Rubpy to have the fastest kinetics and blackberry dye with the lowest kinetics. It can be deduced that the slower the kinetics the more uniform the concentration of the dye across the depth of the substrate.

### Surface Profiling and 3D Imaging of Dye Sensitized TiO_2_ films

The Terahertz scanning reflectometry has shown promise as an important tool for obtaining tomographic information of the surface, subsurface, and interaction of the constituents of a specimen. The Terahertz subsurface imaging was carried out to examine nanoscopic features of TiO_2_ before and after the application of various dyes. Two different TiO_2_ film sizes were scanned: 13000 × 13000 μm^2^ area (Figs 6 and 7) to give a general overview of the sample film and 4 × 4 μm^2^ area (Figs 8 and 9) to obtain detailed features of the film. The surface plots (Figs 6 and 8) and 3D images (Figs 7 and 9) were obtained for blank substrate (TiO_2_ alone); to serve as a control, and pomegranate, blackberry and Rubpy dye diffused TiO_2_ film. The sizes of TiO_2_ films measured in microns are indicated on the coordinate axes.

In Figs 6 and 8, the intensity of reflected light is normalized for the blank TiO_2_ and all the dye sensitized TiO_2_ films for easy visual comparison of the degree to which each sample film reflect light. The intensity of reflected light in Figs 7 and 9 on the other hand is not normalized for all the sample films and thus provide information about the relative intensities of the different parts of a given sample film. It was observed from all the measurements that the intensities of reflected light were not only based on the permeation of the dyes into TiO_2_ film but also due in part to the thickness and morphology of the blank TiO_2_ film. Thin or exposed surfaces of the film produced high intensity of reflected light whereas crests and ridges exhibited low intensity of reflected light.

The reconstructed surface plots (Figs 6 and 8) clearly revealed exposed surfaces and defects present on the dye sensitized TiO_2_ coated FTO glass substrate. A thicker spot near the center of the mesoporous TiO_2_ film created in the process of spinning TiO_2_ paste on the FTO glass slides showed up as an artifact on the surface plot of the blank TiO_2_ and all three dye sensitized TiO_2_ films. Some spots of the TiO_2_ film got detached from the FTO glass on application of the dye resulting in higher intensity of reflected light as seen for Rubpy in Fig. 6. This is indicative of a spin/dye dropping related change of the TiO_2_ film. The thicker spots are less obvious in the 4 × 4 μm^2^ film size scan (Fig. 8) since the scan was performed outside of the main area with artifact.

The artifact was also visible on the 3D images (Fig. 7) and more prominently in Rubpy sensitized TiO_2_ film where the exposed glass in the center of artifact is signified by high intensity of reflected light. A careful examination of 3D reconstructed image of the Rubpy sensitized TiO_2_ film (Fig. 7) shows a complete saturation of the dye in the TiO_2_ matrix. The patches of light blue color may indicate dye evaporated from the surface of the TiO_2_. The permeation characteristic of blackberry dye is similar to that of pomegranate (Figs 6 and 8). The intensity of reflected light is low at the point of application of the dye but increases outwardly.

### Detection of Defects Present of Dye Sensitized TiO_2_ Films

To confirm the unique capability of Terahertz reflectometry to detect flaws present on the dye sensitized TiO_2_ films. A 10 mm × 23 mm portion of 25 mm × 25 mm dye sensitized TiO_2_ films were scanned to detect any defects present on the films. Pomegranate, blackberry and Rubpy dye sensitized solar cell electrodes were scanned and with the measurements, 3-D images, comparable to scanning electron microscopy images, were generated. As displayed in Fig. 10, the cracks present in the TiO_2_ films show up as large peaks on the surface plots due to the greater intensity of reflected light from the exposed glass surface. The magnitude of the peaks correlate with the size of the exposed surface area. Defects are easily noticeable as they reflect more light. As displayed in Fig. 10, the protruding peaks in blackberry dye sensitized TiO_2_ film (Fig. 10a) and pomegranate dye sensitized TiO_2_ film (Fig. 10b) are bigger than that of Rubpy dye sensitized TiO_2_ film (Fig. 10c). These defects are not so much as a result of the dye application as it is the result of the lack of uniformity of the layer of TiO_2_ on the glass substrate. The results demonstrate the unique defect detection capabilities of Terahertz reflectometry and how it is well suited to efficiently detect flaws and malfunctioning areas of solar cells.

## Conclusion

Terahertz spectrometry was used to study and characterize the interaction of two organic dyes (pomegranate and blackberry) and an inorganic dye, Ruthenium (II) polypyridine complex (Rubpy) with mesoporous TiO_2_. A spectroscopic analysis exhibited similar terahertz absorbance peaks for the organic dye sensitized TiO_2_ films which were different from the peaks of the inorganic dye sensitized TiO_2_ films. Diffusion kinetics and concentration distribution profile of the applied dye into the TiO_2_ matrix revealed that Rubpy has the fastest kinetics and blackberry dye has the slowest kinetics. In contrast to the inorganic dye, the organic dyes were uniform through the layers of the mesoporous TiO_2_. Surface profiling and 3D imaging of dye sensitized TiO_2_ films revealed unevenness and flaws present on the film and thus demonstrated the unique defect detection capability of Terahertz reflectometry. Terahertz spectrometry thus provides an effective means to optically measure dye sensitized TiO_2_ electrode properties prior to the fabrication of DSSC.

## Experimental Methods

### Time-domain terahertz spectrometer

Terahertz measurements were carried out using a terahertz time-domain spectrometer with an attached nanoscanner (TeraSpectra, Applied Research & Photonics, and Harrisburg, PA). The spectrometer portion is similar to a setup described previously^5^. Briefly; an electro-optic dendrimer is excited by a pump laser, where continuous wave (CW) terahertz radiation is generated via a mechanism called dendrimer dipole excitation (DDE). This DDE source generates stable terahertz radiation over a range of ~0.1 THz to ~30 THz at room temperature. The measurements are carried out in either a spectrometer mode, for insight into molecular transition within a sample, or in a reflection mode for surface profiling and 3D imaging. The navigation between the two modes is facilitated by a nanoscanner component of the instrument. The nanoscanner position samples in the beam path for transmission measurements and also allows the scanning of samples in three dimensions to probe the inner layers.

**Figure 1:**
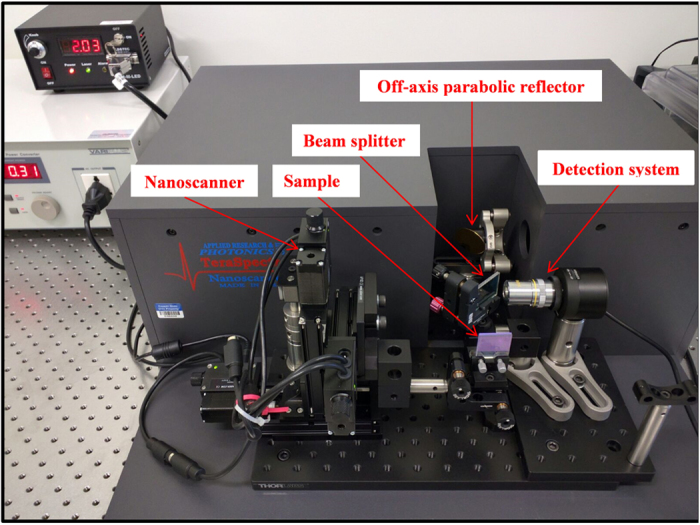
Pomegranate sensitized TiO_2_ slide mounted on the XYZ-stage in the Terahertz probe setup.

**Figure 2:**
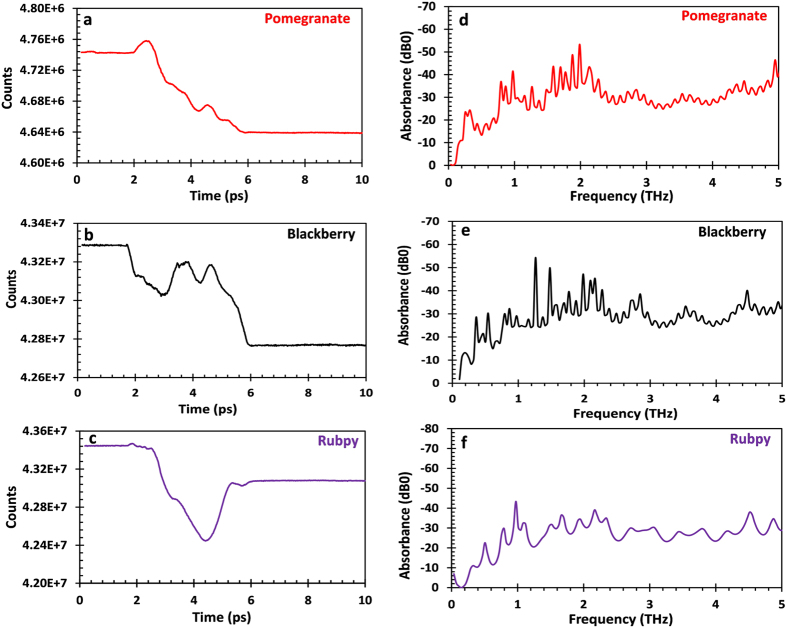
Time-domain temporal signal (**a–c**); its corresponding Fourier transform broadband terahertz absorbance spectra (**d–f**) of pomegranate (**a,d**) blackberry (**b,e**) and Rubpy (**c,f**) dye sensitized TiO_2_ on FTO glass.

**Figure 3:**
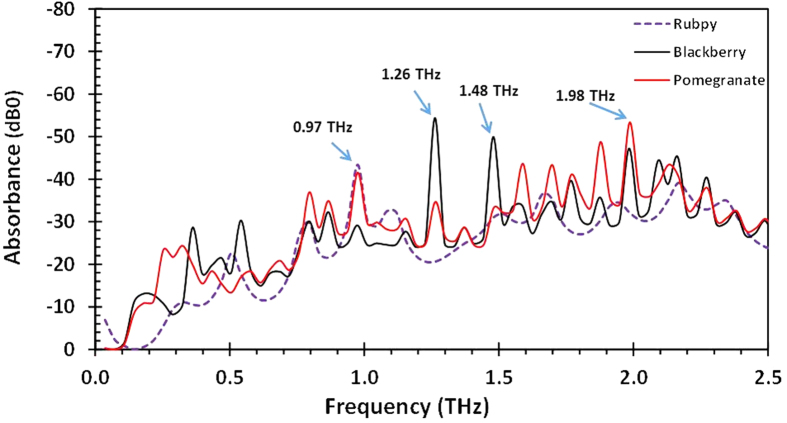
Fourier transform frequency spectra of three different dye sensitized TiO_2_ films (pomegranate, blackberry and Rubpy) showing distinct absorbance characteristics.

**Figure 4:**
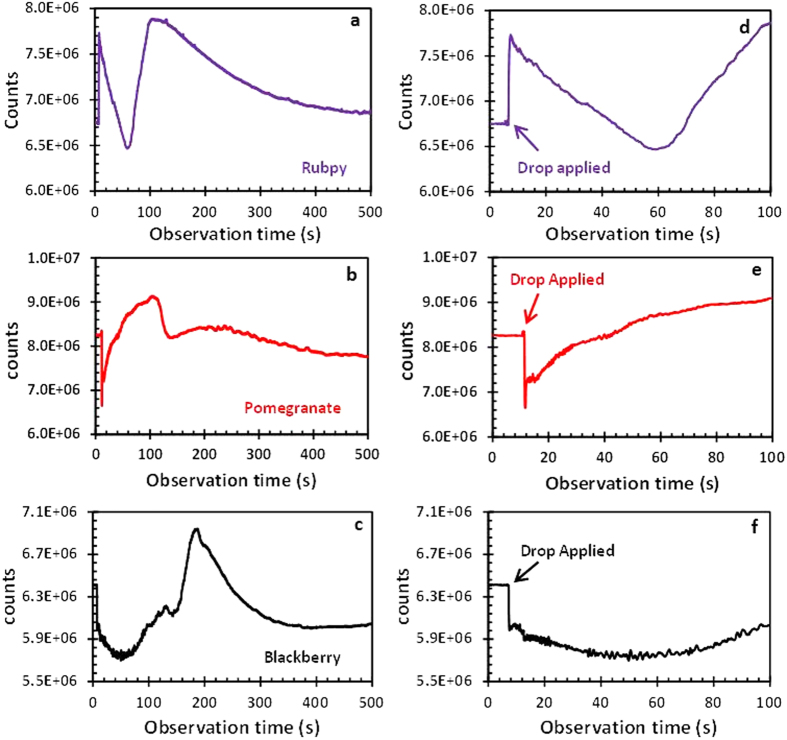
Kinetics of permeation of three different dyes into TiO_2_ films coated on FTO glass: (**a**) Rubpy; (**b**) pomegranate; (**c**) blackberry. Close-up view: (**d**) Rubpy; (**e**) pomegranate; (**f**) blackberry.

**Figure 5:**
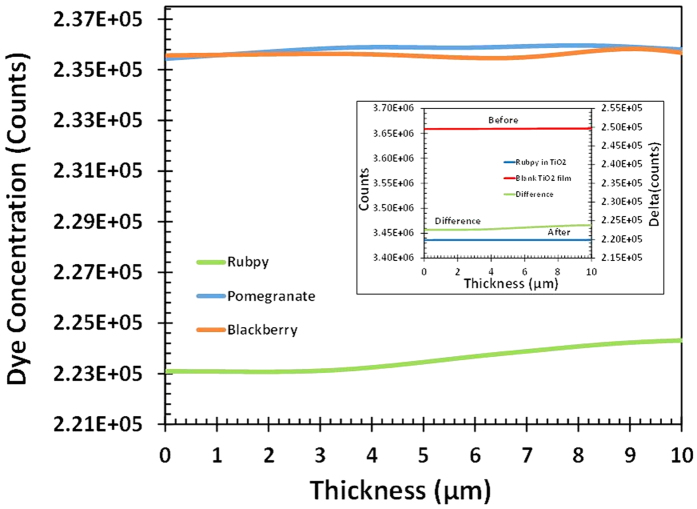
Diffusion characteristics of Rubpy, pomegranate, and blackberry dyes in mesoporous TiO_2_; (inset) Depth scan of Rubpy dye sensitized TiO_2_ film. Top (red) scan of blank TiO_2_ film and bottom (blue) is the scan after the TiO_2_ is saturated with Rubpy dye. The middle curve (green, right axis) is the difference of the top and the bottom curves, indicating the distribution of the Rubpy dye across TiO_2_ film.

**Figure 6:**
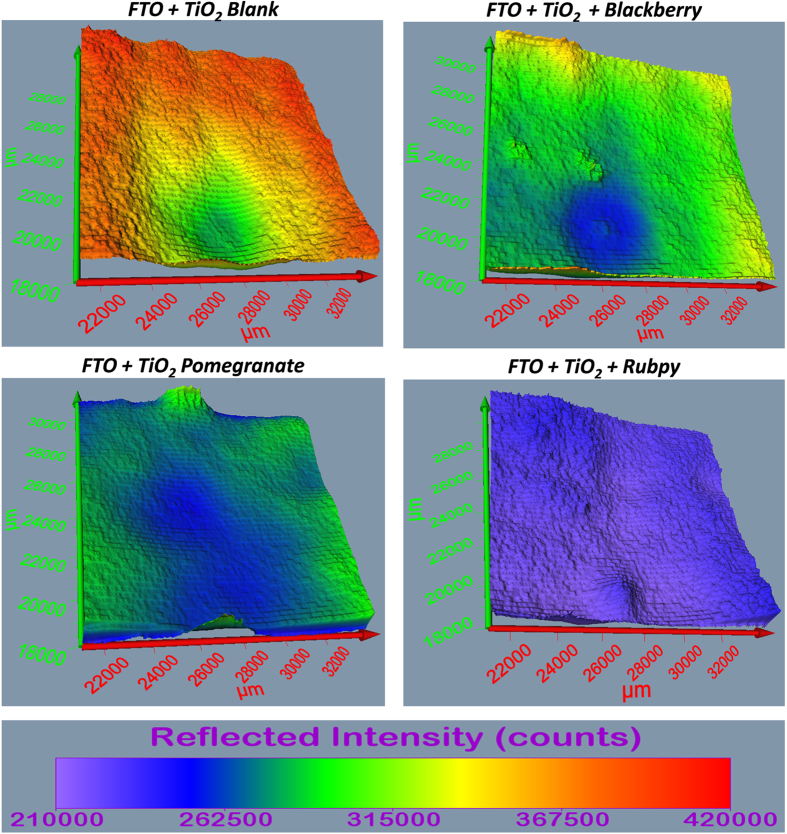
Comparison of the surface plot of blank TiO_2_, blackberry, pomegranate and Rubpy dye sensitized TiO_2_ film coated on FTO glass (Size of film scanned ~13000 × 13000 μm^2^).

**Figure 7:**
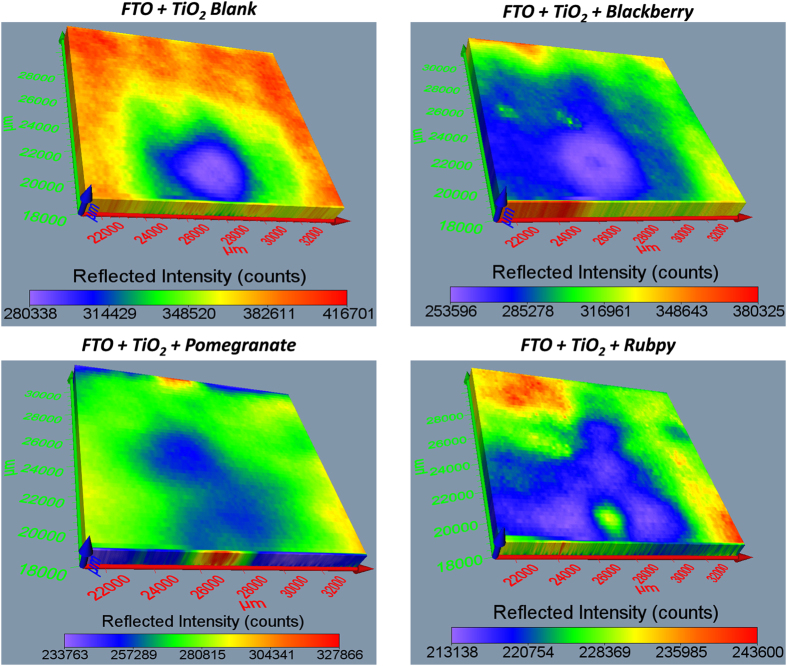
Comparison of the 3D images of blank TiO_2_ blackberry, pomegranate and Rubpy dye sensitized TiO_2_ coated FTO film (Size of sample scanned ~13000 × 13000 μm^2^).

**Figure 8:**
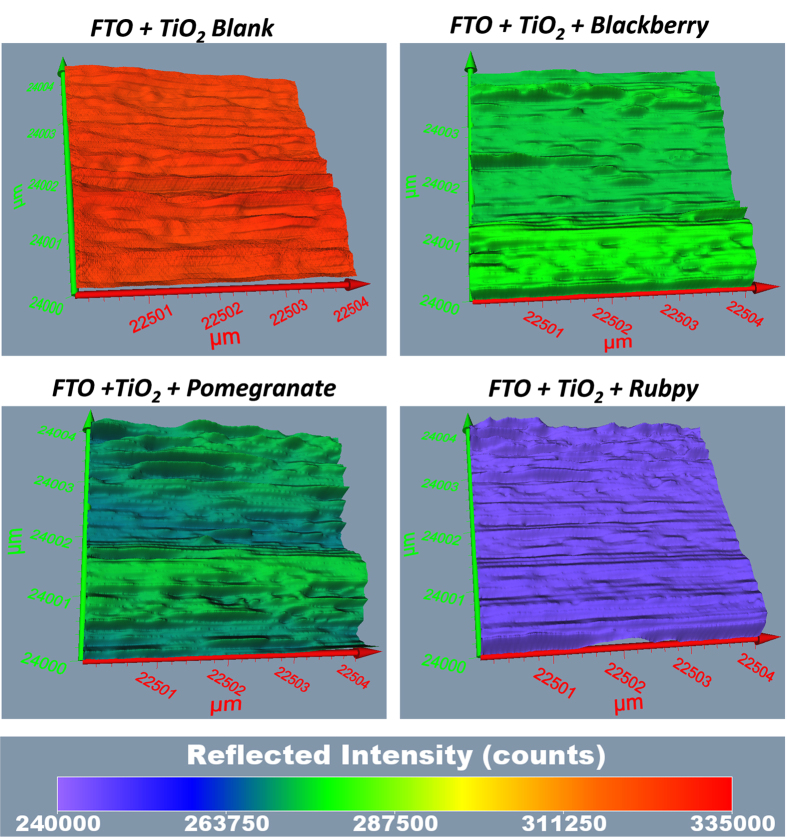
Comparison of the surface plot of blackberry, pomegranate and Rubpy dye sensitized TiO_2_ film coated on FTO glass with their corresponding blank TiO_2_ film coated on FTO glass. (Size of sample scanned ~4 × 4 μm^2^).

**Figure 9:**
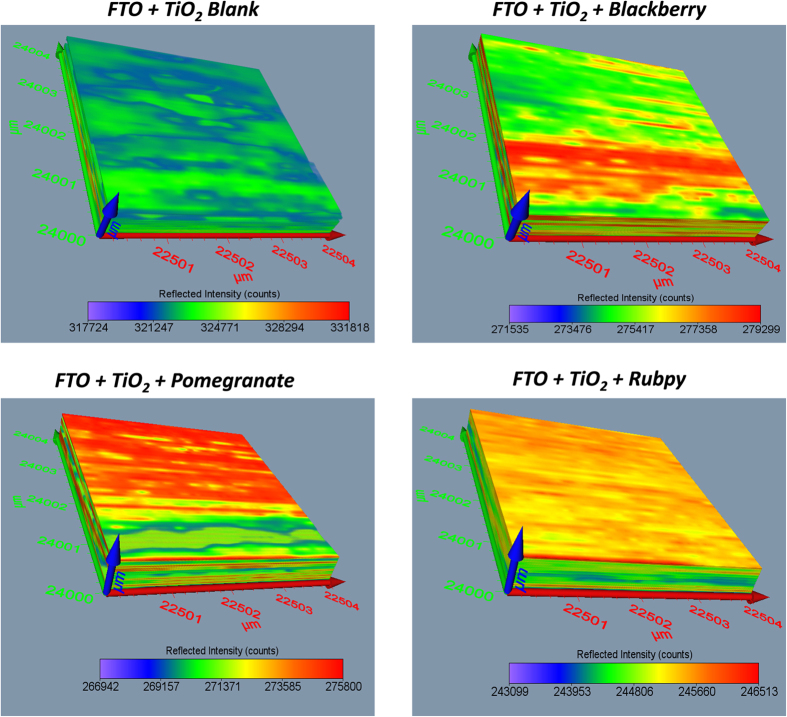
Comparison of the 3D images of blackberry, pomegranate and Rubpy dye sensitized TiO_2_ film coated on FTO glass with their corresponding blank TiO_2_ coated on FTO glass. (Size of film scanned ~4 × 4 μm^2^).

**Figure 10:**
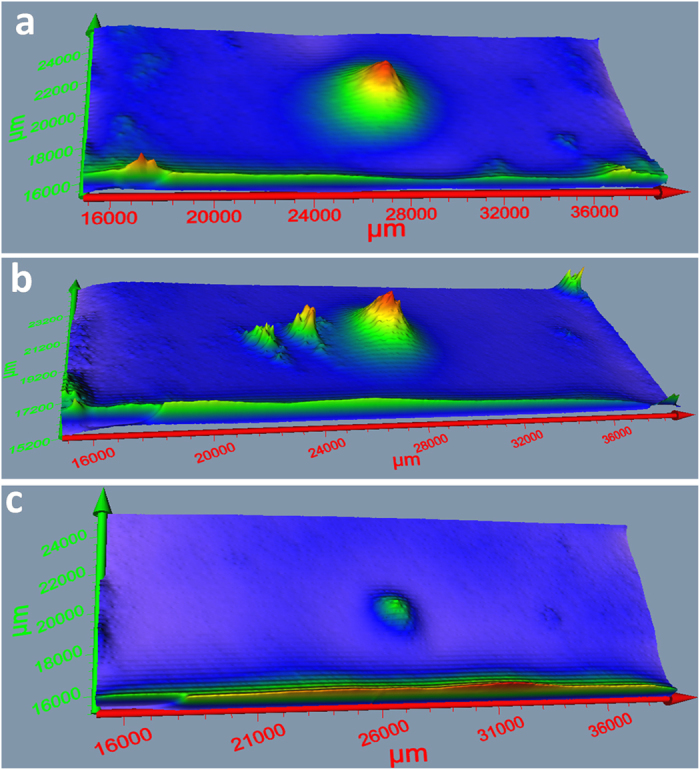
10 mm × 23 mm Surface plots showing defects on dye sensitized TiO_2_ films: (**a**) blackberry; (**b**) pomegranate; (**c**) Rubpy.

Figure 1 shows the TeraSpectra with the reflection module and a sample (pomegranate dye sensitized TiO_2_ film on FTO glass) mounted on the sample holder. During 3D imaging measurements, the terahertz beam first hit the off-axis parabolic reflector and is focused on the sample at a 90 degree angle. The reflected beam from the sample is directed to the detection system via the beam splitter as illustrated in Fig. 1 and Supplementary Figure S1. 3D motion of the sample holder is facilitated by the nanoscanner enabling the interrogation of the reflectance across all the three axis of the sample. Supplementary Figure S1 is a schematic set-up of terahertz scanning reflectometry used for permeation kinetics and concentration profile of the dye in the sub surface of dye sensitized titanium dioxide films.

### Materials

The transparent conductive oxide coated polymer, FTO (1″ × 1″ or 2.5 cm × 2.5 cm) with coating thickness of 4, 000 Angstrom was purchased from Hartford Glass Co. Inc. Titanium Dioxide Power (Degussa P25) was procured from the institute of chemical education. Fresh fruit of pomegranate and blackberry, purchased in Baltimore, were peeled and their dye extracted with a commercial juicer.

### Sample Preparation

The FTO coated conductive glass served as the substrate for the preparation of the samples. TiO_2_ paste, prepared according to a previously published procedure^35^, was spread uniformly across the conductive side of the glass using a spin coater. The TiO_2_ films were subsequently placed on a hot plate with a surface temperature of 380 °C to anneal for over a period of 6 hours. They were then cooled down to room temperature before the application of the freshly prepared dye. For spectral measurements and 3D imaging, the TiO_2_ films were immersed in the dye solution overnight and then rinsed with water and acetone before measurement. Supplementary Figure S4 shows a schematic of the stages involved in the fabrication of the solar cells. For kinetic studies, aliquot 20 μL of the respective dye was measured with a micropipette and dropped directly over TiO_2_ coated FTO film right before measurement.

### Data Analysis

The experimental data obtained in time-domain signal (interferogram or terahertz pulse) were analyzed using the Fourier transform of unevenly sampled data algorithm via commercially available software package AutoSignal^®^ v. 1.7 by SeaSolve Software Inc. The surface plots and 3D images were generated by means of voxler^®^ 3 visualization software from Golden Software Inc. The data for kinetic studies were analyzed via Microsoft Excel 2013.v.

## Additional Information

**How to cite this article**: Ghann, W. *et al*. Interaction of Sensitizing Dyes with Nanostructured TiO_2_ Film in Dye-Sensitized Solar Cells Using Terahertz Spectroscopy. *Sci. Rep*. **6**, 30140; doi: 10.1038/srep30140 (2016).

## Supplementary Material

Supplementary Information
