# Supplementary material for: Interaction of Sensitizing Dyes with Nanostructured TiO2 Film in Dye-Sensitized Solar Cells Using Terahertz Spectroscopy
Source: Sci Rep. 2016 Jul 22;6:30140. doi: 10.1038/srep30140 (PMC4957082; doi:10.1038/srep30140)
Supplement: Supplementary Information [file srep30140-s1.pdf]

Supplementary information for: "Interaction of Sensitizing Dyes with Nanostructured TiO_2 film in Dye-Sensitized Solar Cells Using Terahertz Spectroscopy"

William Ghann[1], Aunik Rahman[2], Anis Rahman[2], Jamal Uddin[1]*

[1]Center for Nanotechnology for Department of Natural Sciences, Coppin State University, 2500 W. North Avenue, Baltimore, MD 21216. [2]Applied Research & Photonics, 470 Friendship Road, Suite 10, Harrisburg, PA 17111. Correspondence and requests for materials should be addressed to J.U. (email:juddin@coppin.edu)

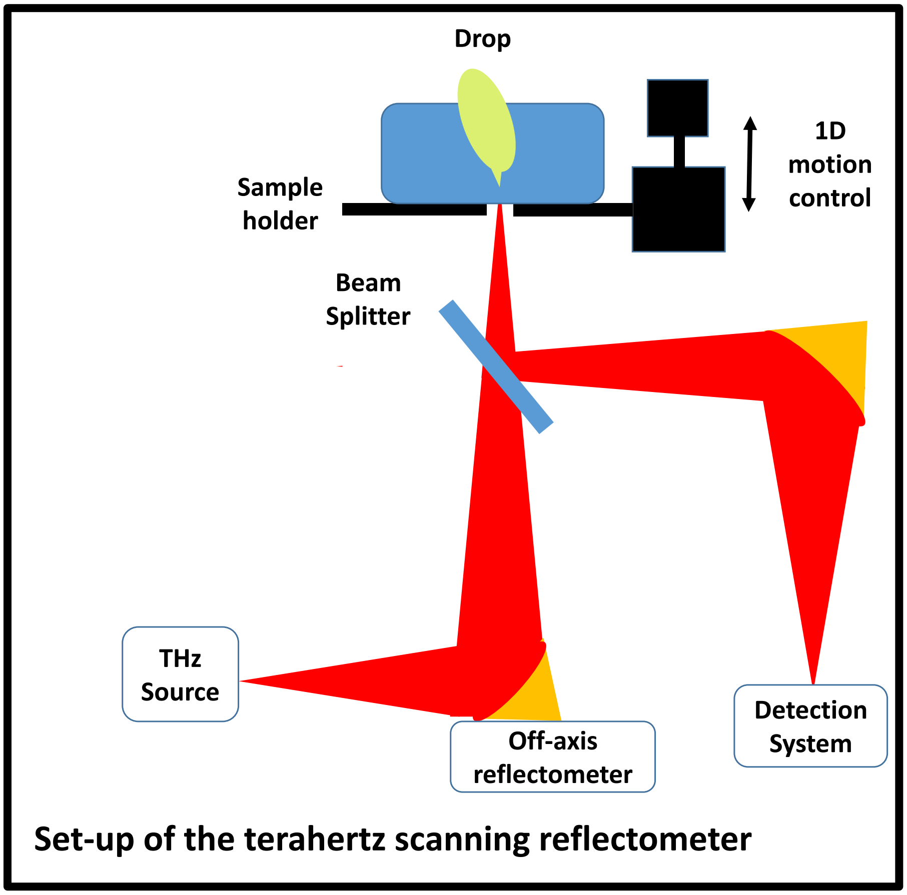

Figure S 1: Set-up of the terahertz scanning reflectometer

Terahertz Scanning Reflectometer

During 3D imaging measurements, the terahertz beam first hit the off-axis parabolic reflector and is focused on the sample at a 90 degree angle. The reflected beam from the sample is directed to the detection system via the beam splitter as illustrated in Supplementary Figure S 1. 3D motion of the sample holder is facilitated by the nanoscanner enabling the interrogation of the reflectance across all the three axis of the sample.

Surface Plots and 3D Imaging of $\mathbf{10 \times 10\ \mu m^2}$ Area of Dye Sensitized $\mathbf{TiO_2}$ Films

Supplementary Figure S 2 exhibits another close up of the respective substrates over $10 \times 10\ \mu m^2$ area. The corresponding range of intensities as described by the legend in increasing values are approximately 276,000 – 286,000 for Rubpy dye sensitized TiO_2 films, 298,000 - 311,000 for pomegranate dye sensitized TiO_2 films and 379,000 – 395,000 for blackberry dye sensitized TiO_2 films. The blank sample had the highest intensities of reflected light with 38, 000 – 40,000 counts. Since a higher value means a greater reflected intensity, blackberry dye sensitized TiO_2 films, accordingly, reflect more light than all the other dye sensitized TiO_2 films followed by pomegranate dye sensitized TiO_2 films and Rubpy dye sensitized TiO_2 films, respectively. If Rubpy dye sensitized TiO_2 films reflect light the least then it could be assumed that it either absorb or transmits most of the T-ray incident on it. This observation is consistent with results obtained from the kinetics studies carried out.

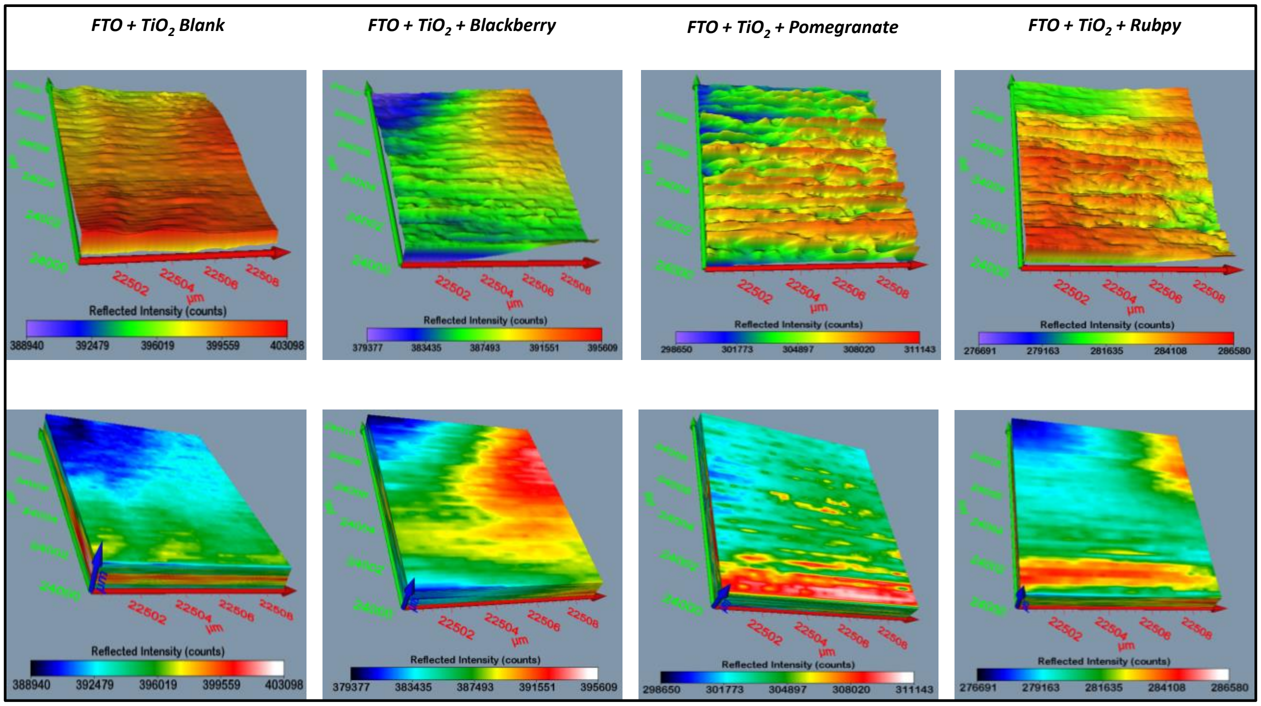

Figure S 2: Comparison of the surface plot (top) and 3D images (bottom) of blackberry, pomegranate and Rubpy dye sensitized TiO_2 coated FTO film with their corresponding blank TiO_2 coated FTO film. (Size of sample scanned ~ $10\times10\ \mu m^2$)

Reflection scan of Blackberry dye sensitized TiO_2 film

The TeraSpectra uses terahertz radiation generated from electro-optic dendrimer source to scan and produce images of samples. Supplementary Figure S 3 shows the raw data of 3D imaging of Blackberry dye sensitized TiO_2 film. The measurement is carried out in the reflection mode in which the nanoscanner tool attached to the time-domain terahertz spectrometer scans the sample for surface profiling and 3D imaging. The intensity of reflectance measured in counts over the distance scanned are first acquired and subsequently processed to obtain surface plots and 3D images. Blackberry dye sensitized TiO_2 film was scanned from 21, 250 µm to 34, 000 µm with respect to the position of the laser thus covering a distance of 12,750 µm on one dimension of the TiO_2 film. There is little or no absorption at bare spots of the TiO_2 films resulting in high

intensity of reflected light as seen in the middle of portion of the scan in Supplementary Figure S 3.

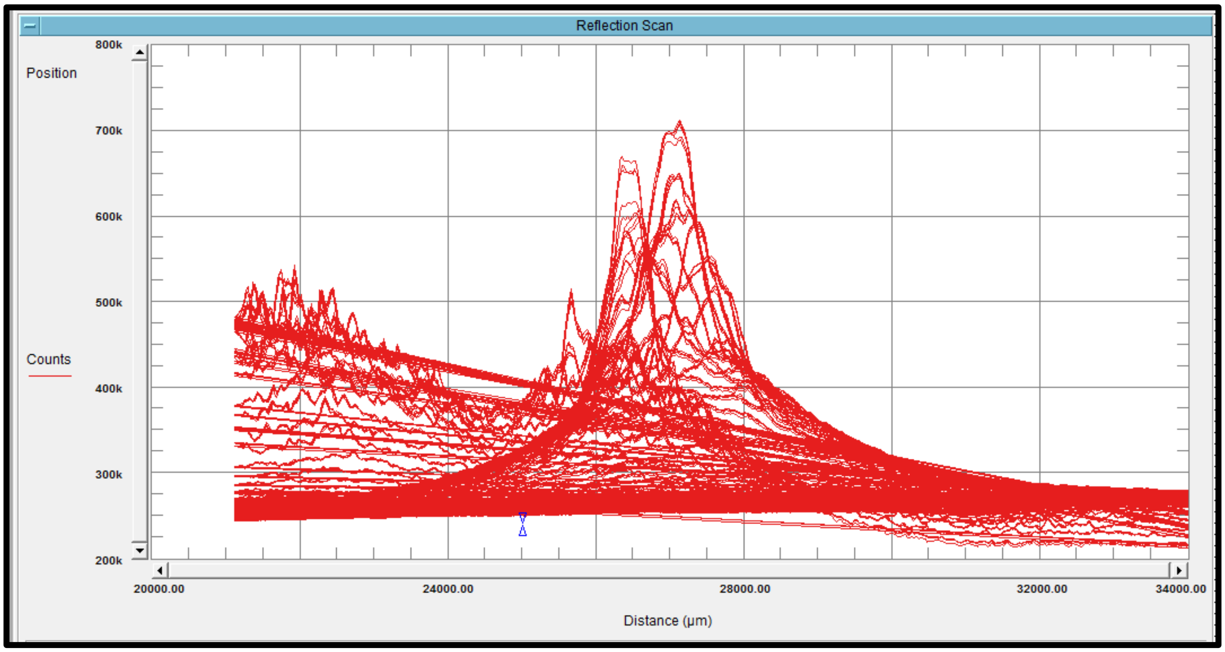

Figure S 3: Reflection Scan of blackberry dye sensitized solar cell

Preparation of Dye Sensitized TiO_2 films

Fluorine doped tin oxide on glass (FTO) of size 25 mm x 25 mm was used as a substrate for the preparation of the samples. Scotch tape was applied to one side of the FTO glass substrate to mask ~5 mm of the conductive layer. The FTO glass substrate was subsequently spin coated with TiO_2 paste (Supplementary Figure S 4). The spin coating process was followed by the removal of the scotch tape and sintering of the TiO_2 coated FTO glass at 450°C for 90 minutes on a hotplate. The average thickness of the TiO_2 film measured using Field Emission Scanning Election Microscope (FESEM) was approximately 9µm. For surface profiling and 3D imaging, dye was applied on the TiO_2 film as shown in Supplementary Figure S 4b and allowed to adsorb unto the TiO_2 over a period of 12 hours at room temperature. The dye-adsorbed TiO_2 film was washed

with water and acetone and dried to obtain the dye sensitized FTO/TiO_2 film as shown in Supplementary Figure S 4c. For the studies on the diffusion characteristics, the dyes were directly applied to FTO/TiO_2 film right before measurements were carried out.

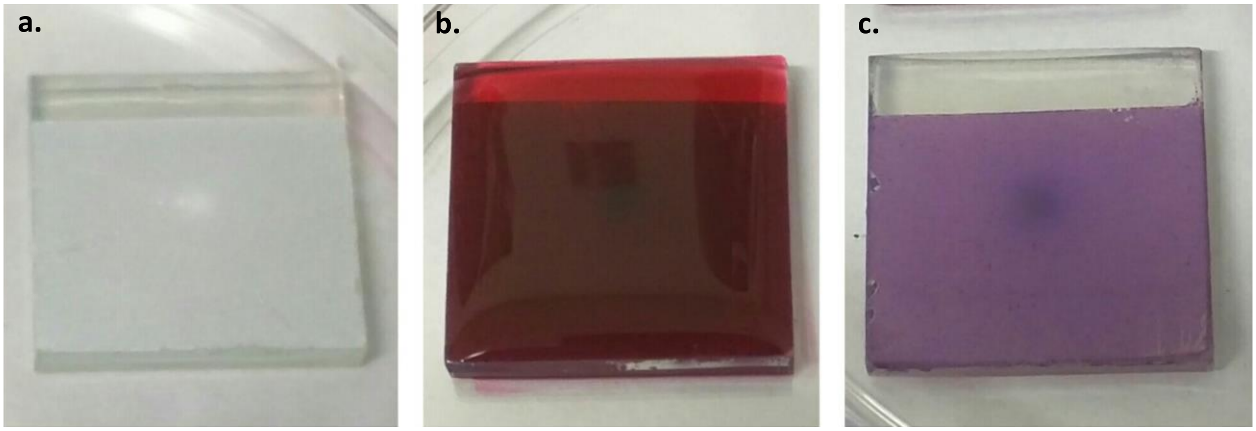

Figure S 4: Sample Preparation; (a) blank TiO2 coated FTO film; (b) pomegranate dye solution getting adsorbed unto TiO_2 coated film; (c) pomegranate sensitized TiO_2 film

Spectroscopic Studies of Blank TiO_2 films

Time-domain temporal signal, also known as interferogram, of the blank TiO_2 (Supplementary Figure S 5) was measured for comparison with the three dye sensitized TiO_2 slides utilized in the studies. To obtain the absorbance values (Figure S 5b), Fourier transform algorithim of unevenly sample data, otherwise known as Lomb periodogram, was deployed for the analysis of the experimental data.

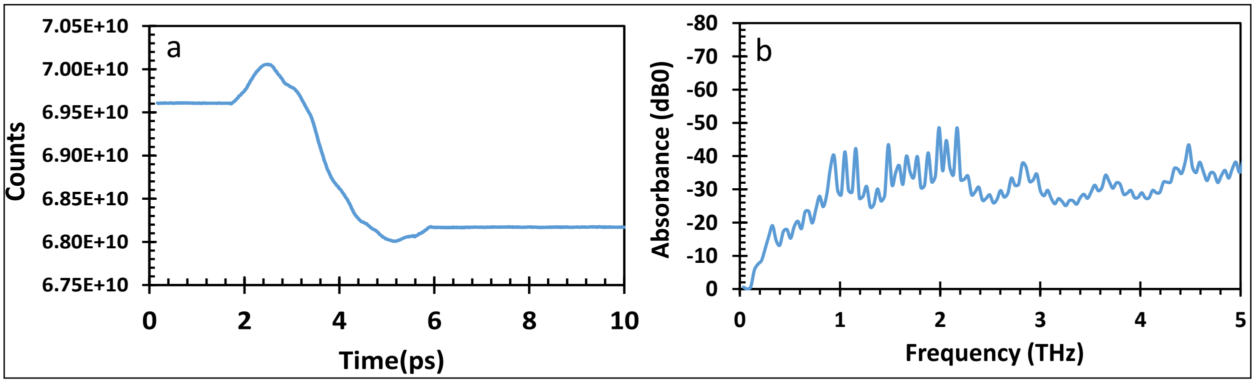

Figure S 5: Time-domain temporal signal (left) and it corresponding Fourier transform broadband terahertz absorbance spectra (right) of blank titanium dioxide
